# Platelets express adaptor proteins of the extrinsic apoptosis pathway and can activate caspase-8

**DOI:** 10.1371/journal.pone.0244848

**Published:** 2021-01-11

**Authors:** Nadine Goelz, Julia J. M. Eekels, Milica Pantic, Christoph T. Kamber, Oliver Speer, Francesca D. Franzoso, Markus Schmugge

**Affiliations:** 1 Division of Haematology and Children’s Research Center, University Children’s Hospital Zurich, Zurich, Switzerland; 2 Division of Transfusion Medicine, University Hospital Greifswald, Greifswald, Germany; 3 Institute for Laboratory Medicine, Hospital Thurgau AG, Münsterlingen, Switzerland; Columbia University, UNITED STATES

## Abstract

**Background:**

Apoptotic pathways in platelets are important for their survival and function. Platelet apoptosis may be involved in the pathogenesis of immune thrombocytopenia (ITP), an autoimmune-mediated disease. In contrast to the intrinsic apoptosis pathway, not much is known about the extrinsic pathway mechanisms in platelets.

**Objectives:**

To investigate the expression of proteins involved in the extrinsic apoptosis pathway, including the death receptors, adaptor and regulator proteins in human platelets. To determine a possible trigger of the extrinsic apoptosis pathway in platelets.

**Methods:**

To investigate the expression of key markers of the extrinsic pathway we used targeted immunofluorescence and flow cytometry assays. To study their expression and interaction we performed Western blotting and co-immunoprecipitation. Treated platelets with different apoptosis triggers were subjected to flow cytometry.

**Results:**

We could identify the protein expression of the pro-apoptotic proteins TRADD (Tumor Necrosis Factor Receptor type 1- Associated DEATH Domain protein), TRAF2/5, (TNF Associated Factor) and DEDAF (Death Effector Domain- Associated Factor), FADD (Fas-Associated protein with death domain) as well as the anti-apoptotic proteins DJ-1 (Deglycase 1) and c-FLIP in human platelets. ABT-737 treatment induced a disruption in the co-localization of DJ-1 with FADD. Platelets treated with ABT-737 showed an activation in caspase-3 and -8. The exposure to TNF (Tumor Necrosis Factor), FasL (Fas ligand), and TWEAK or to plasma derived from ITP patients, did not lead to changes in caspase-3 and -8 activation in platelets.

**Conclusions:**

Human platelets express some proteins of the extrinsic apoptosis pathway which can be modulated only by ABT-737 treatment. However so far, no other apoptosis trigger or interaction with an external receptor have been yet identified.

## Introduction

Apoptosis is the major cellular mechanism, which regulates the cell life span and the removal of damaged or infected nucleated cells. In nucleated cells two major apoptosis pathways are known: the intrinsic apoptosis pathway, regulated by pro- and anti-apoptotic members of the Bcl-2 family at the level of mitochondria, and the extrinsic pathway, that is initiated by the interaction between death ligands and death receptors of tumor-necrosis factor receptor (TNF-R) superfamily and is regulated by death inducing signaling complexes (DISC) [1]. Each of these pathways can be triggered by specific stimuli that leads to activation first of initiator caspases, caspase-8 and/or -10 (extrinsic pathway) and caspase-9 (intrinsic pathway) and then of executor caspase-3, -6, and -7. A third cell-mediated apoptosis pathway, known as the perforin/granzyme pathway, can induce cell death via granzyme A and B [2]. To date, several DISC pathways and their apoptotic adaptor proteins are well characterized in nucleated cells, e.g. the Fas Ligand (L)/Fas receptor (R), TNF-α/TNFR1, Apo3L/Death receptor 3 (DR3), Apo2L/DR4, and Apo2L/DR5 [3].

It is known that platelets contain apoptotic regulators, including members of the Bcl-2 protein family and downstream effectors, e.g. caspases-3, -8-, -9, and -10 [4], as well as a fully functional intrinsic apoptosis pathway [5]. In contrast to nucleated cells, not much is known about the extrinsic apoptosis pathway in anucleated platelets. One report showed mRNA expression of the death receptor ligand TRAIL (TNF-Related Apoptosis Induced Ligand), death receptors TNF-R1, DR3, DR4, and DR5, and the adaptor proteins TRADD and RIP (Receptor-Interacting Protein) in human platelets [6]. However, at the protein level, only the TRAIL receptor DR5 could be identified [7], and the death receptor FasL in activated platelets [8].

Apoptotic inducers, e.g. ABT-737 and A23187 have been shown to trigger apoptotic like events via the intrinsic apoptosis pathway in platelets [5, 9]. Interestingly, Mutlu et al. demonstrated that treatment of platelets with BH3-only mimetic drug ABT-737 or calcium ionophore A23187 can activate caspase-8 through the mitochondrial pathway without interacting with the death receptors [10]. Moreover, ABT-737 treatment had been shown to induce regression of solid tumors [11], but at the same time ABT-737 therapy limits platelet survival and can even induce platelet apoptosis [12].

Previous studies from our group and from others found evidence that platelet apoptosis can play a role in immune thrombocytopenia (ITP), an autoimmune-mediated disease causing low platelet count and eventually bleeding [5, 9, 13]. It was suggested that, beside ABT-737, also plasma of ITP patients can induce an increase in PS exposure in ITP platelets or in normal platelets incubated with autologous CD3+ lymphocytes [14]. However, it is still unknown which components of the ITP plasma could trigger apoptosis caspase cascade in platelets. A possible mechanism was shown by Zhang et al. who discovered a cell-mediated lysis of autologous platelets with CD8+ cytotoxic T cells (CTLs) as effector cells in chronic idiopathic ITP patients mediated by the perforin/granzyme pathway through CTLs [15].

In the present study, we first aimed to investigate the protein expression of the apoptotic adaptor proteins TRAF2, TRAF5, c-FLIP (cellular FLICE (FADD-like IL-1β-converting enzyme)-inhibitory protein), DEDAF, FADD, and DJ-1 in human platelets. Second, we explored if DJ-1 can interact and co-localize with FADD in human platelets. Next, we studied the effects of TNF, FasL, TWEAK and ITP plasma treatment compared to ABT-737 or A23187 stimulation in platelets by investigating the caspase -3/7 and -8 activation, as well as the protein expression of DJ-1 and FADD.

## Materials and methods

### Blood sampling and platelet concentrates

This study was approved by the local ethical committee (Cantonal Ethics Committee Zurich, Switzerland) and written informed consent was obtained from all healthy blood donors as well as from ITP patients, or their legal guardians. All patients were recruited at the Children’s Hospital Zurich, Switzerland.

Venous blood samples for immunofluorescence staining (IF) or flow cytometry analyses were taken from healthy donors who had not ingested any anti-platelet medication for at least two weeks and had a normal blood count. Samples were collected freshly in EDTA (final concentration of 1.6mg EDTA/ml blood) or citrate anticoagulant (final concentration 10.5mM), depending on the assay. Platelets concentrates (PCs) were obtained from the local blood bank (Blood Donation Service Zurich) and contained 2.4 x 10^11^ platelets/unit with a maximum leukocyte contamination of 1 x 10^6^ leukocytes/unit. PCs were used for Western blot and co-immunoprecipitation (co-IP) analyses.

Platelet-rich plasma (PRP) of each subject group was collected after 10min centrifugation at 140g at room temperature (RT) without brake. Afterwards, plasma samples were obtained at 2min centrifugation at 13000g at RT. Plasma of each subject group was stored until further use at -80°C.

### Patient characteristics

All patients fulfilled the criteria for primary ITP [16]. In total, we recruited 20 acute ITP patients at diagnosis and 22 acute ITP patients treated with intravenous immunoglobulin (IVIg) with median platelet count: 6.35 **±** 5.79 x10^9^/L; median age: 6.16 **±** 3.8 years; gender ratio: 10 females (F) / 12 males (M)). For comparison, 16 chronic ITP patients (median platelet count: 77.60 **±** 86.46; median age: 6.01 **±** 2.86 years; gender ratio: 8F/8M) were studied. Chronic ITP patients were identified as patients with a persistent low platelet count (<100 x 10^9^/L), which lasted longer than one year [16]. In addition, we included 13 healthy pediatric controls with a median platelet count of 263.5 **±** 125.5 x10^9^/L and a median age of 8.6 **±** 5.3 years (4F/ 9M).

### ABT-737 treatment of washed platelets

Preparations of washed platelets were carried out as described previously [5]. Briefly, PRP was obtained by centrifugation at 140g for 10min at RT without brake. Supernatant was removed and transferred into another tube supplemented with anticoagulant citrate dextrose solution (ACD) [5]. PRP was centrifuged at 1200g at RT for 12min. The platelet pellet was re-suspended in Tyrodes-Hepes buffer [5] containing 2 mM CaCl2, 0.02% EGTA and 0.35% albumin. After each resuspension step, platelets were left at RT for 10min and supplemented with 72ul ACD per ml wash solution before centrifugation at 800g. The second washing step included Ca^2+^-free Tyrodes-Hepes buffer without EGTA and the final suspension medium was a Tyrodes-Hepes solution supplemented with 0.35% BSA (TAH), 0.05U/ml apyrase and 2.5mM GPRP.

Platelet apoptosis was induced by stimulating the platelets derived from healthy donors with ABT-737 (final conc. 3μM) for 2h at 37°C. Unstimulated platelets were kept for 3h at 37°C. After the incubation time, platelets were centrifuged at 13000g and platelet pellet was lysed on ice with a thrombocyte lysis (TC) lysis buffer + protease inhibitor (50mM Tris/HCl (pH 8), 150mM NaCl, 1% Tween, and 1mM EDTA). Protein concentration was measured with the Pierce™ BCA protein assay kit (ThermoFisher Scientific, cat. 23227) according to the manufacturer’s protocol, and a microplate fluorescence reader Tecan, Tecan Infinite M200 PRO (Switzerland). Platelet lysates were frozen and stored at -20°C until further use.

### Immunofluorescence staining and confocal microscopy

IF staining was performed using washed platelets obtained from ETDA blood. Washed platelets were diluted 1:2 in TAH buffer and 100μl were loaded onto a cytospin disposable sample chamber as previously described [17, 18]. Samples were centrifuged at 800g for 4min at RT to immobilize the platelets onto glass slides. To ensure a strong binding of the platelets on the glass slide platelets were air dried for 5min and fixed for 20min at RT by using Fix/Perm buffer (BD Biosciences). Afterwards, platelets were washed and blocked with PBS + 5% Fetal bovine serum (FBS) for 20min at RT. All incubation steps were performed in a humidity chamber to avoid drying. For IF staining platelets were washed 3 x with PBS + EDTA (final concentration 100nM). Incubation with the primary antibody was done overnight (o/n). Platelets were washed and incubated with the secondary antibody (1:1000 dilution) in PBS + 2% FBS for 60min at RT. Samples were covered with DAPI containing mounting media (Life Technologies). Slides were stored at 4°C until further use. Detailed antibody list is summarized in S1 Table.

For confocal microscopy, platelets were characterized using CD41 staining. Confocal laser scanning microscopy was performed using a Leica SP8 upright confocal or the Leica SP8 inverse confocal microscope with an objective magnification of × 63 (Immersion of 1.3). Acquired images were imported into Imaris software (version 7.7.2) and analyzed using ImageJ software 1.45 (NIH, MD, USA).

### Flow cytometry

Flow cytometric analyses were done using washed platelets obtained from EDTA whole blood. To analyze intrinsic proteins washed platelets were fixed and permeabilized in Fix/Perm (BD Biosciences) solution for 20min at RT. Afterwards, platelets were washed with washing buffer (10x Perm/Wash Buffer; BD Biosciences) and centrifuged for 10min at 500g. All washing steps were performed with a washing buffer from BD and followed by 500g centrifugation for 10min at RT. To block nonspecific binding, blocking buffer (PBS+5% FBS) was added to the platelets and incubated for 30min at RT. Supernatant was removed and the primary antibody was added for 30min at RT. After removing the supernatant, secondary antibodies (1:1000 dilution) were diluted in PBS + 2% FBS and incubated for 30min at RT. The platelets surface marker CD42a- or b- PE was added and incubated for 30min at RT. Detailed antibody list is presented in S1 Table. For each sample, 10’000 platelets were identified as CD42a- or CD42b-positive events. All samples were acquired using a FACS Canto II flow cytometer (Becton Dickinson, Rotkreuz, Switzerland). Data was analyzed with FlowJo software (version 10.0).

### Co-immunoprecipitation and Western blot analyses

PCs were lysed and protein concentration was measured as above. 500–1500μg protein extracted from PCs was used to perform the co-Immunoprecipitation (co-IP). 1.5mg (50 μl) of magnetic Dynabeads Protein A/G (Thermo Fisher Scientific, cat. 1003D) was pre-incubated o/n at 4°C under continuous mixing on a vertical rotator with a desired antibody (4μg) additionally PBS-Tween20 (final Tween concentration 0.01%) was added. The antibody-bead-complex was washed according to the manufacturer’s protocol with PBS-Tween20 and re-suspended in PBS. Antibody-bead-complex was incubated with the PC lysate (protein concentration of 500μg) in PBS for 2h at RT on a vertical rotator. The antibody-protein-bead-complex were washed and eluted using NuPage LDS Sample Buffer (Invitrogen) + DTT (50mg) for 10min at 70°C on a shaker (400rcf). The eluted sample was analyzed by gel electrophoresis, Western blot analyses or stored at -20°C until further use.

Western blot analyses were performed using washed PRP. We cannot rule out some platelet activation, as we could not assess for platelet activation. Fresh or frozen lysates were either directly used or thawed on ice. PRP samples were separated via gel electrophoresis (Bolt™ 4–12% Bis-Tris Plus Gel; Invitrogen) and transferred to PVDF membranes in cooled transfer Buffer (20x NuPage Transfer Buffer; Novex) at 90V for 60min. Membranes were blocked for 1h at RT in blocking buffer (TBS + 5% FBS), and after each antibody incubation the membrane was washed with washing buffer (10x TBS; 1M Tris/HCl, 5M NaCl) and TWEEN to a final concentration of 0.2%. For detection, the primary antibody was diluted 1:250–1:1000 (depending on the antibody used) in TBS with 2% BSA and incubated overnight at 4°C. The HRP-conjugated secondary antibody was added (diluted 1:5000 or 1:10000 in TBS with 2% BSA) for 1h at RT. Membranes were visualized with chemiluminescence imaging (Chemi Doc MP Imager; BioRad) using ECL substrate (GE Healthcare). The detailed antibody list is showed in S1 Table.

### Incubation experiments with plasma from ITP patients and ligands

Blood samples were collected in citrate anticoagulant (final concentration 10.5mM). We used plasma samples from 3 ITP patients and 2 healthy controls, stored at -80°C. Washed platelets were incubated for 2h at 37°C with either plasma derived from ITP patients (ITP plasma), autologous plasma derived from the healthy platelet donor (autologous plasma) or plasma derived from a healthy control (healthy plasma).

Additionally, healthy washed platelets were stimulated with A23187 (final concentration of 0.5μM) to induce platelet apoptosis. After stimulation, washed platelets were stained with PE-conjugated CD42a, a specific platelet marker. For active caspase-3, -8 and -9, we used the FLICA substrate from Millipore; FAM-DEVD-FMK (caspase-3), FAM-LETD-FMK (caspase-8) or FAM-LEHD-FMK (caspase-9). Washed platelets were analysed by flow cytometry (see section above).

Ligand treatment was done by stimulating the platelets for 3h with either TNF (100ng/μl), Fas (100ng/μl) or TWEAK (100ng/μl) at 37°C. The ligands were a gift from Wei-Lynn Wong, University of Zurich, Switzerland. After stimulation, washed platelets were stained with PE-conjugated CD42a, a specific platelet marker and for Annexin V conjugate, a marker for platelet activation. To detect active caspase-3, we used the FLICA substrate FAM-DEVD-FMK (Millipore) according to the manufacturer’s manual. After 30min of incubation at RT washed platelets were centrifuged at 500g for 10min at RT. After removing the supernatant 500μl of PBS was added and the washed platelets were analysed by flow cytometry, as mentioned above.

### Multiplex granzyme assay and data analysis

The MILLIPLEX Multiplex Assay was run according to manufacturer's instructions (MerckMillipore, Germany), using paramagnetic beads (6.5 μm diameter). For subsequent assessment of endogenous granzyme A and B concentrations in our samples, we used the MILLIPLEX MAP Human CD8+ T cell (HCD8-6015) kit. EDTA or citrate plasma samples were incubated overnight at 4°C on a rotation shaker (18h, 750rpm). Plates were washed on a hand-held magnetic block according to the manufacturer’s protocol.

Data were acquired on a validated and calibrated Bio-Plex 200 system (Bio-Rad) and analyzed with Bio-Plex Manager 5.0 software (Bio-Rad) with a detection target of 50 beads per region, low RP1 target for CAL2 calibration and recommended doublet discriminator (DD) gates of 8000–15,000 MILLIPLEX kits. We excluded from the standard curve any points with %CV >25% and those with accuracy outside of 70–140% of expected range. The analysis software was used to fit a curve to this set of reliable standards data using five parameter logistic regression with default automated weighting (all fitted to ≥ 6 points).

### Statistical analysis

Statistical analysis was performed using GraphPad Prism Software Version 6.00 (GraphPad, Software CA, USA). Continuous variables were compared using one-way ANOVA non-parametric Kruskal-Wallis test, followed by multiple comparisons tests to compare the mean ranks between groups. Normally distributed data was analyzed using t-Tests or One-way ANOVA. Data are presented as Standard Error of the Mean (SEM). Significance is shown as p < 0.033 (*), p < 0.0021 (**), p < 0.0002 (***), p < 0.001 (****).

## Results

### Identification of proteins of the extrinsic apoptosis pathway in platelets

**Fig 1:**
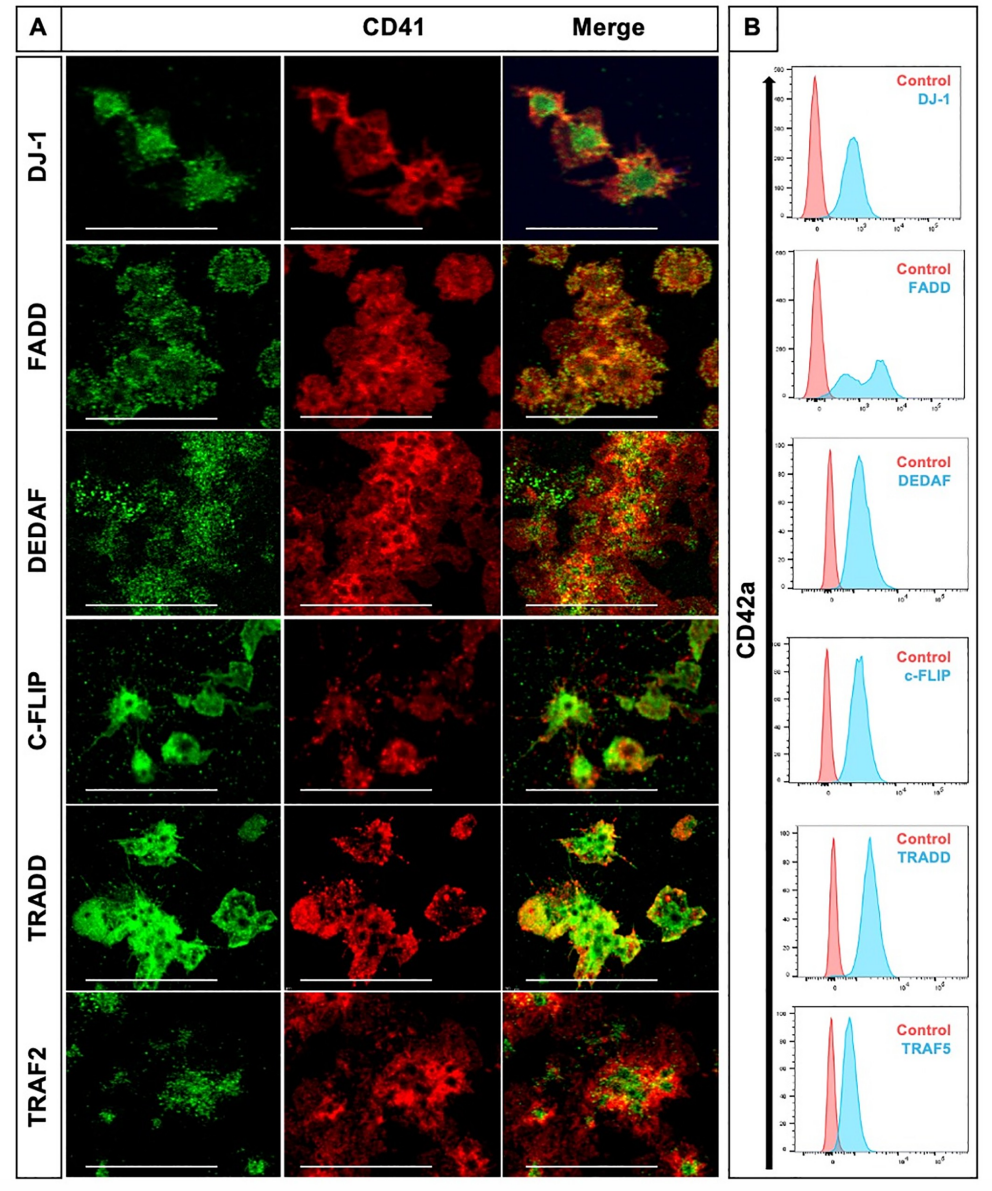
Pro- and anti-apoptotic adaptor proteins of the extrinsic pathway of apoptosis are expressed in human platelets. (A) Representative images of platelets co-stained for CD41 (red) with either DJ-1, FADD, TRAF2, TRADD, c-FLIP or DEDAF (all displayed in green). CD41 stains for integrin αIIb (GPIIb), a transmembrane glycoprotein expressed on the platelet surface and on megakaryocytes. Shown is one confocal plane, objective: 63x glycerol with a numerical aperture of 1.3. Images were processed by using confocal laser scanning microscope SP8 (Leica). Scale bar 20μm. (B) Identification of DJ-1, FADD, TRAF5, TRADD, c-FLIP, and DEDAF by flow cytometry. Results are presented as CD42a positive events. Importantly, TRAF2 (green) was only identified by confocal microscopy (A) and TRAF5 (blue) was only detected by flow cytometry analysis (B). We used IgG isotype as control.

To investigate the protein expression of key markers of extrinsic pathway such as apoptotic adaptor proteins TRAF2, TRAF5, c-FLIP, DEDAF, FADD, TRADD and DJ-1 in human platelets, we used confocal microscopy and flow cytometry analyses. We were able to identify the expression of pro-apoptotic proteins FADD, TRADD, DEDAF and the anti-apoptotic proteins c-FLIP and DJ-1 in washed human platelets by both methods (Fig 1). TRAF2 was identified only by IF whereas TRAF5 only by flow cytometry with the antibodies mentioned in S1 Table.

### Co-localization and possible interaction of DJ-1 and FADD in human platelets

**Fig 2:**
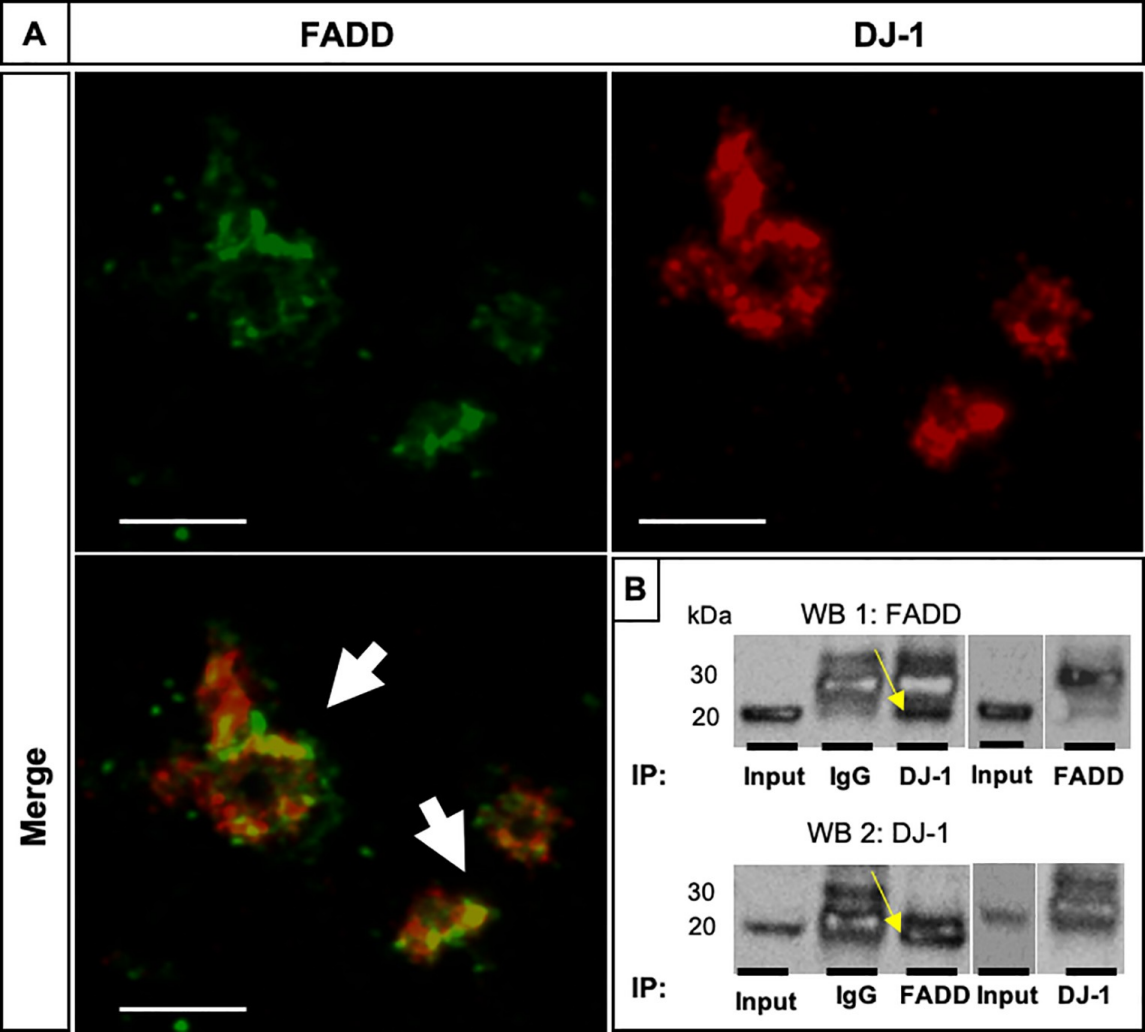
FADD and DJ-1 co-localize and possibly interact in human platelets. (A) Representative confocal images show the co-localization of FADD (green) and DJ-1 (red) resulting in a yellow stain (white arrows). Shown is one confocal plane, objective 63x glycerol with a numerical aperture of 1.3. Images were processed by using confocal laser scanning microscope SP8 (Leica). We also calculated a colocalization correlation coefficient Pearson’s R value of 0.88 with Image J software. Scale bar 10μm. (B) co-IP of FADD and DJ-1 in human PCs. As control the whole platelet lysate was immunoblotted (Input) and IgG controls were used to determine unspecific binding of the protein to the antibody (n = 3). Immunoblot 1 (WB 1 top): FADD probed; Immunoblot 2 (WB 2 bottom): DJ-1 probed.

We evaluated whether DJ-1 and FADD proteins could share the same subcellular localization in healthy, non-apoptotic platelets. FADD and DJ-1 showed a moderate co-localization assessed by IF staining in confocal images (Fig 2A, white arrows, with Pearson’s correlation coefficient, R = 0.88 and Mander’s overlap coefficient of 0.48, also shown individually in S3–S5 Figs). As this could be a good indicative of a possible interaction and binding to each other, we performed co-IP experiment using PCs (Fig 2B). After a pulldown assay of FADD and DJ-1 in PCs we compared the pulldown samples with the IgG controls and the input samples by IP (Fig 2B). When FADD was used for the pulldown, a clear band was visible, corresponding to the molecular weight of DJ-1 (Fig 2B, WB 1 top, yellow arrow). In contrast, in the immunoblot for DJ-1, FADD protein expression signal was observed at the same height as the light chain of the IgG antibody, which means that the light chain of the IgG antibody was probably masking the putative signal for FADD (Fig 2B, WB 2 bottom, yellow arrow). As controls we used the whole platelet lysate (Input), IgG controls and the antibodies which specifically immunoprecipitated either FADD or DJ-1. These results suggest a possible interaction of DJ-1 and FADD in human platelets.

### ABT-737 induces caspase-8 activation and can modulate the co-localization of DJ-1 and FADD

**Fig 3:**
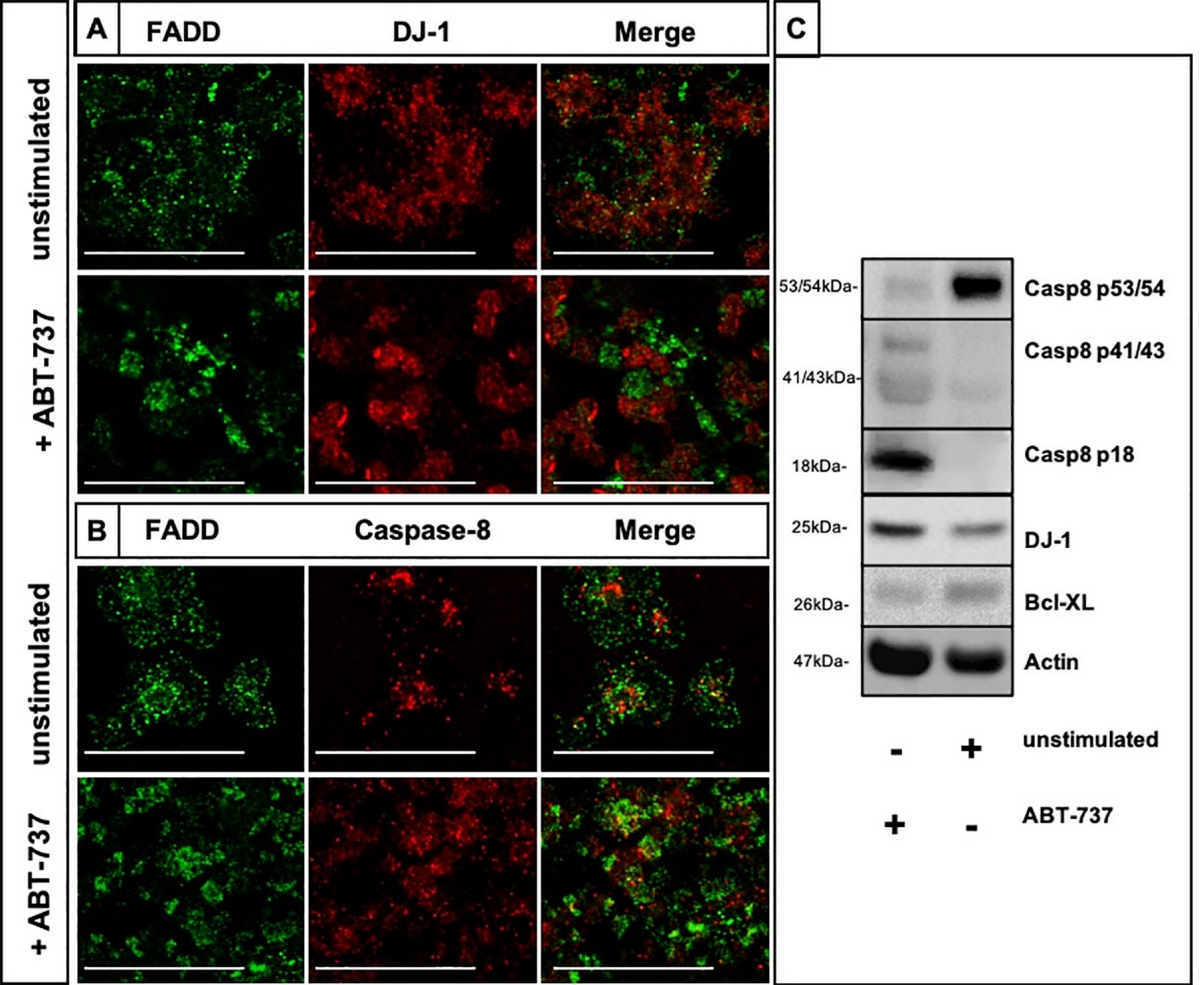
ABT-737 exposure disrupts the co-localization of DJ-1 with FADD and activates caspase-8 in platelets. (A) Immunofluorescence co-staining of unstimulated and ABT-737 stimulated platelets for FADD (green) and DJ-1 (red). Merged images show that the moderate co-localization of DJ-1 and FADD (yellow, upper image) is disrupted in ABT-737 stimulated platelets (lower image). (B) Immunofluorescence co-staining of unstimulated and ABT-737 stimulated platelets for FADD (green) and caspase-8 (red). Merged images show increased co-localized expression of FADD and caspase-8 in ABT-737 stimulated platelets. Results are shown as one confocal plane, objective 63x glycerol with a numerical aperture of 1.3. Images were processed by using confocal laser scanning microscope SP8 (Leica). Scale bar 20μm. (C) Caspase-8 (procaspase-8 p53/54, first cleavage fragments p41/43, and the active p18 form), DJ-1 and Bcl-XL expression was assessed by Western blotting in ABT-737 treated platelets (lane 1) and in unstimulated cells (lane 2). Platelets were treated for 2h at 37°C. A representative immunoblotting image was selected (n = 3).

We investigated the effects of the exposure of ABT-737, an apoptosis- and thrombocytopenia-inducing drug, on the expression of FADD and DJ-1 as well as on the caspase-8 activity in human platelets. Platelets were stimulated with ABT-737 *in vitro* for 2h. Confocal imaging depicted a moderate co-localization of FADD (green) and DJ-1 (red) (Fig 3A) and FADD and caspase-8 (Fig 3B) in the unstimulated platelets, while a clear separation of FADD and DJ-1 proteins (Fig 3A) and increased merged caspase-8 and FADD expressions were observed in ABT-737-stimulated platelets (Fig 3B, merged yellow dots). ABT-737 stimulation induced a clear activation and cleavage of caspase-8 (p18 form) (Fig 3C and S1 Fig), as well as significantly decreased protein expression levels of DJ-1 in comparison to unstimulated platelets (Fig 3C and S1 Fig).

### TNF, FasL, and TWEAK ligand treatment does not induce apoptosis in human platelets

**Fig 4:**
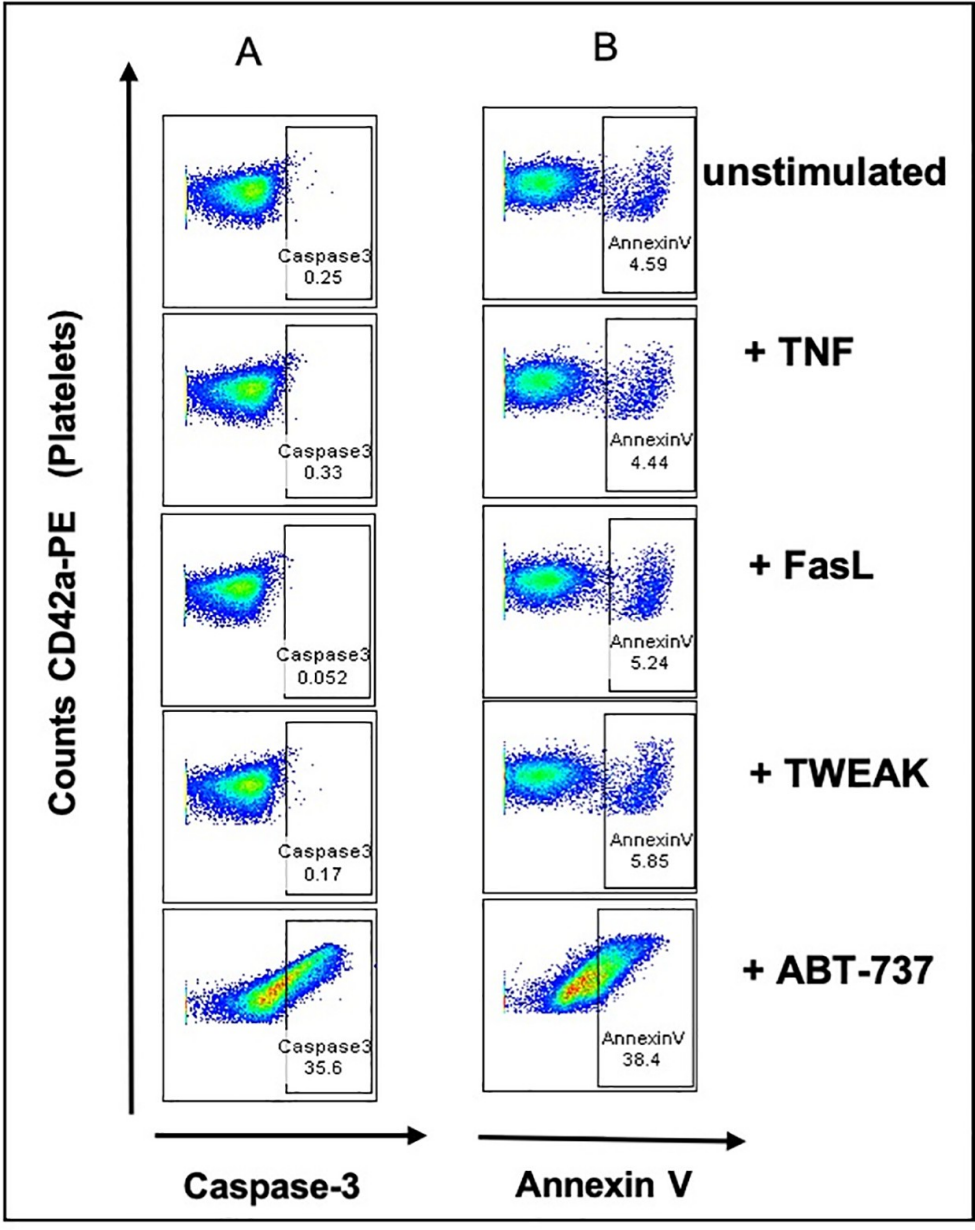
TNF, FasL, and TWEAK treatment of human platelets does not show caspase- 3/7 activation and PS exposure. Flow cytometry of washed platelets treated with TNF, FasL, TWEAK or ABT-737. (A) Platelets were labeled with CD42a-PE (Y-axis) and caspase- 3/7 positivity is shown as fluorescence intensities of FLICA positive events, measured as green fluorescent signal (X-axis). Percentages show double CD42a-PE and caspase-3/7 positive events. CD42a-PE positive only events (platelets) are shown on the left side of the graph. (B) PS exposure is shown as fluorescence intensities of AnnexinV (APC) positive platelets (Y-axis). Percentages show double CD42a-PE and AnnexinV positive events. CD42a-PE positive only events (platelets) are shown on the left side of the graph.

To investigate if treatment with receptor ligands of the extrinsic pathways could induce apoptosis in platelets, we incubated washed healthy platelets with the death receptor ligands TNF, FasL, and TWEAK. ABT-737 treatment was used as positive control and the unstimulated samples as negative control. Upon ABT-737 treatment, we could clearly observe an activation of caspase-3 (35.6% of fluorescence intensity (FI) of FLICA positive platelets in Fig 4A) and the phosphatidylserine (PS) exposure measured as Annexin V positivity in stimulated platelets (38.4% of FI in Fig 4B), compared to unstimulated CD42a positive platelets. Interestingly, the treatment with TNF, FasL, and TWEAK did not show any effect on the PS exposure and on the caspase-3 activation in platelets (Fig 4A and 4B).

### Plasma from ITP patients does not activate caspase-3/7 and -8 in healthy platelets

**Fig 5:**
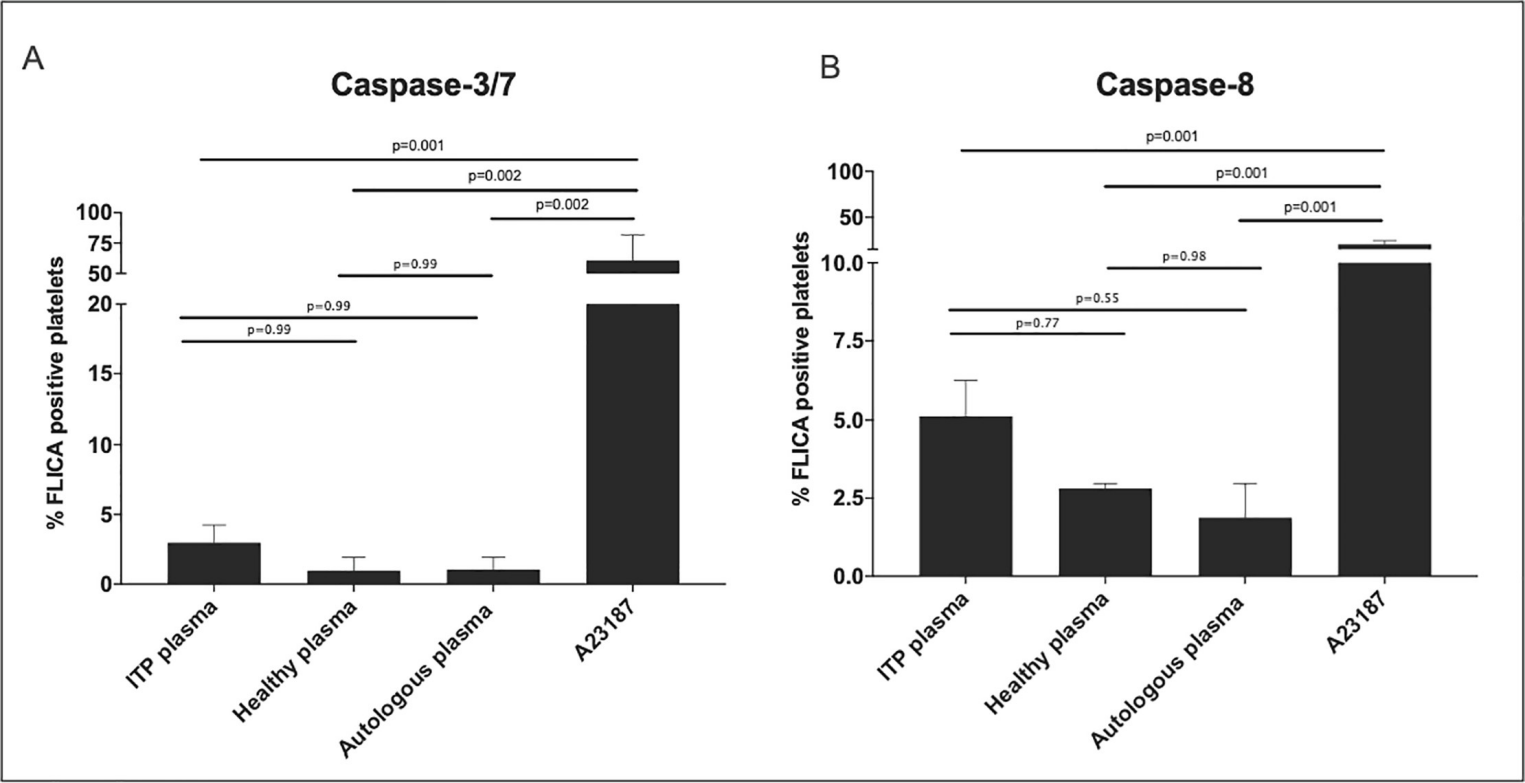
ITP plasma treatment of platelets derived from healthy donors show no significant caspase- 3/7, and -8 activation. Flow cytometry analyses of healthy platelets treated with ITP (n = 3), healthy (n = 2) and autologous plasma (n = 2) and A23187 (n = 2). Healthy platelets were incubated for 2h with ITP plasma at 37°C. FLICA% platelets are: (A) FLICA reagent, FAM-DEVD-FMK, irreversibly bound to active caspase- 3/7, and (B) FLICA reagent, FAM-LETD-FMK, irreversibly bound to active caspase- 8 (B), both measured as green fluorescent signal. Autologous plasma was used as a negative control. Statistical analyses were performed using one-way ANOVA followed by multiple comparisons tests to compare the mean ranks between groups. Data are presented as Standard Error of the Mean (SEM).

To determine if the treatment with plasma from ITP patients could induce apoptosis in platelets, we challenged washed platelets from healthy controls (n = 2) with plasma derived from ITP patients (n = 3) and investigated the activation of caspase- 3/7 and -8. As a positive control, we used platelets incubated with the apoptosis inducer A23187 [5]. Autologous plasma treatment was used as a negative control. After 2h of challenge, no significant changes in caspase- 3/7 and -8 activation were observed when comparing to healthy platelets treated with healthy plasma or to autologous plasma (Fig 5). In contrast, platelets treated with A23187 showed a significant increase of caspase- 3/7 and -8 activation compared to healthy plasma samples (caspase-3/7 *p* = 0.0026 (Fig 5A); caspase-8 *p* = 0.0019 (Fig 5B)), plasma from ITP patients (caspase-3/7 *p* = 0.0010 (Fig 5A); caspase-8 *p* = 0.0015 (Fig 5B)) or autologous plasma samples (caspase-3/7 *p* = 0.0026 (Fig 5A); caspase-8 *p* = 0.0014 (Fig 5B)).

## Discussion

**Fig 6:**
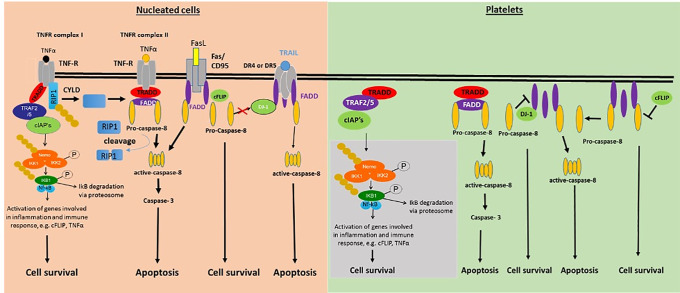
Schematic overview of pro- and anti-apoptotic proteins of the extrinsic apoptosis pathway identified in human platelets (right) in comparison with nucleated cells (left). In nucleated cells, several death receptors are known, e.g. TNFRI/II, Fas/CD95, DR 1–5, which could not be yet identified in human platelets. Grey box: adaptor-proteins of the extrinsic apoptosis pathway identified by our group and others, e.g. TRADD, TRAF2/5, cIAP1/2, FADD, DJ-1, NEMO, IKK1/2, NFκB, and NFκB1.

The current study explores key proteins expression and possible extrinsic pathway triggers in platelets. We identified in platelets for the first time regulator proteins, e.g. DJ-1, FADD, c-FLIP, and adaptor proteins, e.g. DEDAF, TRAF2/5, and TRADD, whereas no death receptor protein expression was detectable (as summarized in Fig 6). In contrast to ABT-737 platelet apoptosis drug inducer, the treatment with ITP plasma, TNF, FasL or TWEAK showed no effects in the activation of caspase-8 and-3, suggesting a selective caspase-8 induction.

Interestingly, Li et al. identified mRNA levels of TRAIL, TNFR1, DR3, DR4, and DR5, TRADD and RIP in fresh platelets [6], whereas on the protein level in PCs, DR4 and DR5 were not found [17]. In other studies, TNFR1 expression has been investigated in human platelets only by flow cytometry [19], whereas FADD expression was detected in mouse platelets only by immunoblotting [20]. Treatment of platelets with anti-Fas antibodies did not induce caspase-8 activation [18], but activated platelets express the membrane bound ligand FasL, and can activate caspase 3/7 in human neuroblastoma cells and mouse embryonic fibroblasts [8]. Although our first screening part of the study showed the presence of some extrinsic adaptor proteins, we could not identify the expression of any death receptor ligand. This suggests that extrinsic pathway apoptosis in platelets cannot be induced in the classical way, by death receptors, as in nucleated cells.

DJ-1 was shown to play a significant role in protecting nerve cells from oxidative stress and to be associated with Parkinson’s disease pathogenesis. Additionally, DJ-1 also protects cells against UV radiation, suggesting that this protein could play an important role for cell survival [21, 22]. We were able to identify that DJ-1 could co-localize with FADD at subcellular level in non-apoptotic platelets. Furthermore, our data demonstrated that in apoptotic conditions, like ABT-737 exposure, FADD co-localized with activated caspase-8. These results suggest that the ABT-737-induced apoptotic effects may lead to a loss of interaction capacity between FADD and DJ-1, probably due to DJ-1 competition with activated caspase-8 for binding to FADD, as in nucleated cells. DJ-1 inhibited TRAIL-induced apoptosis by blocking the pro-caspase-8 recruitment to FADD in HCT116 cells [23]. Based on this study, DJ-1 acted as an anti-apoptotic protein by binding to FADD and blocking its interaction with pro-caspase-8 after external apoptotic stimuli. Previous work of our group showed that the pretreatment of platelets with UCF-101, an inhibitor of the intrinsic apoptosis pathway, could diminish the activation of caspase- 3/7 and -9 after ABT-737 stimulation. Moreover, as the activation of caspase-8 activation was still present, even after ABT-737 stimulation, it was suggested that ABT-737 can act on both apoptosis signaling pathways in platelets [5]. Consistent with this, Mutlu et al. showed that ABT-737 indirectly activates caspase-8, through the mitochondria‐initiated caspase activation signaling cascade, with no interaction with the death receptors on the cell surface [10]. Activation of caspase-8 after ABT-737 treatment has been also reported in mouse platelets [20].

As a limitation of our study, the platelet activation could not be assessed before the immunofluorescence and immunoblot analysis, therefore the platelet activation may influence some of the protein expressions that were analysed. However, we normalized the protein levels to the internal controls and performed adequate statistical analysis of the data (Figs 2 and 3 and S1 Fig).

With respect to the function of the FasL and TRAIL death ligands, contrasting observations were reported in platelets: while one study was unable to induce platelet apoptosis by the use of anti-Fas antibodies, another group showed that the death receptor ligand TRAIL can induce platelet apoptosis [7, 18]. In our study, treating platelets with TNF, FasL, and TWEAK did not result in caspase- 3/7, -8, and -9 activation in platelets. This suggests that platelets could have a different triggering mechanism for extrinsic pathway, respectively for caspase-8 activation. It was previously reported that platelet apoptosis can be mediated by their surface receptors PAR-1, GPIIbIIIa and GPIbα rather by using death receptors (reviewed by [1]).

Platelet apoptosis could play a role in the pathogenesis of ITP, an autoimmune disease characterized by accelerated platelet destruction. Our group previously found activated caspase -3, -8, and -9 in platelets of pediatric ITP patients [13]. In the present study we could not observe caspase -3/7, -8, and -9 activation in healthy platelets treated with plasma from ITP patients, indicating that the plasma content alone cannot induce apoptotic events in platelets. These results stand in agreement with other reports. Recently, a study described that, although healthy platelets incubated with plasma derived from ITP patients showed an increase in the inner mitochondrial depolarization, PS exposure on the platelet surface and an increase in PAC-1 binding, no caspase-3, -8 and -9 activation were found [19]. Investigations by Goette et al. demonstrated that caspase-3 activity and PS exposure remained unchanged after challenging normal platelets with ITP plasma. Nevertheless, they observed an enhanced inner transmembrane potential Δψm depolarization, concluding that an antibody mediated cell toxicity was the underlying mechanism of apoptotic mechanisms in ITP [14]. Co-culturing healthy platelets with autologous CTLs and subsequent treatment with plasma derived from chronic ITP patients showed apoptosis induction in healthy platelets [15], suggesting that a cell-meditated interaction of CTLs or other immune cells are needed to induce apoptosis in platelets. It is possible that ITP specific molecules could be released into the plasma by immune cells, e.g. granzymes and perforin [20] and they could penetrate the platelet cell membrane without using any death receptors on the cell surface. However, we could not detect any significant changes for granzyme A- and B-plasma levels in our acute (n = 20) and chronic (n = 16) ITP patient cohort (S2 Fig).

Our data indicate for the first time that platelets express some members of the extrinsic apoptosis pathway which can also interact. It remains still unclear the role of these extrinsic proteins and triggering of caspase activation in platelets, that seems to be different to nucleated cells. These findings could be used as a basis for future mechanistic studies to fully understand platelet apoptosis and how extrinsic pathway contributes to it.

## Supporting information

S1 Fig Protein levels quantification of Caspase-8, DJ-1 and Bcl-XL in ABT-737 stimulated versus unstimulated cells. Quantification of Caspase-8, DJ-1, Bcl-XL and β-Actin protein levels was determined by calculating integrated densities of each of the Western blot bands from the Fig 3C, using the BioRad Image Lab 5.2.1. software. The integrated intensity is proportional to the amount of the antibodies on the membrane. Data was normalized to actin protein levels and is presented as a percent of actin, as loading control (representing 100%). Statistical analysis was performed using multiple t-Tests.
(TIFF)

S2 Fig Granzyme A and B plasma levels show no significant differences in ITP patients compared to healthy controls. Multiplex assay was used to detect (A) granzyme A and (B) granzyme B concentrations (ng/ml) in plasma samples. (A) Granzyme A is slightly increased in ITP patients, but no significant changes between the plasma level of acute ITP before (n = 20) and after IVIg treatment (n = 22), and in chronic ITP patients (n = 16) were observed. (B) Granzyme B plasma levels remained unchanged. Plasma samples were analyzed with a BioPlex 200 reader. Crtl = healthy controls, acute ITP = ITP patients at diagnosis without treatment, acute ITP+IVIg = ITP patients 24-48h after IVIg treatment, and chronic ITP = ITP patients which have a persistent platelet count (<100x 10^9^/L) which lasts longer than one year after initial diagnosis. Statistical analyses were performed using one-way ANOVA followed by multiple comparisons tests to compare the mean ranks between groups. Data are presented as Standard Error of the Mean (SEM). Significance is shown as p < 0.033 (*), p < 0.0021 (**), p < 0.0002 (***), p < 0.001 (****).
(TIFF)

S3 Fig Cellular localization of FADD in platelets. Representative confocal image showing the FADD immunofluorescence staining (green). Shown is one confocal plane, objective 63x glycerol with a numerical aperture of 1.3. Images were processed by using confocal laser scanning microscope SP8 (Leica). Scale bar 10μm.
(TIF)

S4 Fig Cellular localization of DJ-1 in platelets. Representative confocal image showing the DJ-1 immunofluorescence staining (red). Shown is one confocal plane, objective 63x glycerol with a numerical aperture of 1.3. Images were processed by using confocal laser scanning microscope SP8 (Leica). Scale bar 10μm.
(TIF)

S5 Fig Colocalization of FADD and DJ-1 in platelets. Representative confocal image showing the FADD (green) and DJ-1 immunofluorescence staining (red). Shown is one confocal plane, objective 63x glycerol with a numerical aperture of 1.3. Images were processed by using confocal laser scanning microscope SP8 (Leica). Scale bar 10μm.
(TIF)

S6 Fig Original blots data. Original blots from Figs 2B and 3C and S2 Fig are shown.
(PDF)

S1 Table Antibodies used for flow cytometry, co-immunoprecipitation, Western blotting and IF staining. (DOCX)
