# Supplementary figures and images for: Platelets express adaptor proteins of the extrinsic apoptosis pathway and can activate caspase-8
Source: PLoS One. 2021 Jan 11;16(1):e0244848. doi: 10.1371/journal.pone.0244848 (PMC7799768; doi:10.1371/journal.pone.0244848)

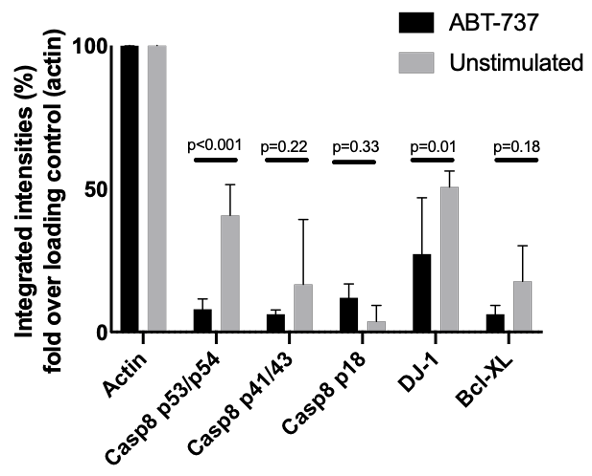

Supplement: S1 Fig — Quantification of Caspase-8, DJ-1, Bcl-XL and β-Actin protein levels was determined by calculating integrated densities of each of the Western blot bands from the Fig 3C, using the BioRad Image Lab 5.2.1. software. The integrated intensity is proportional to the amount of the antibodies on the membrane. Data was normalized to actin protein levels and is presented as a percent of actin, as loading control (representing 100%). Statistical analysis was performed using multiple t-Tests. (TIFF) [file pone.0244848.s001.tiff]

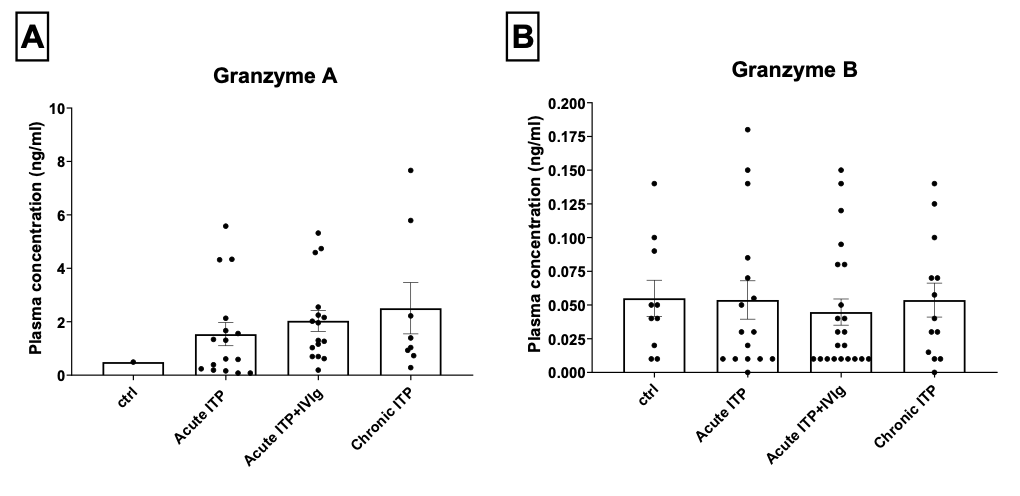

Supplement: S2 Fig — Multiplex assay was used to detect (A) granzyme A and (B) granzyme B concentrations (ng/ml) in plasma samples. (A) Granzyme A is slightly increased in ITP patients, but no significant changes between the plasma level of acute ITP before (n = 20) and after IVIg treatment (n = 22), and in chronic ITP patients (n = 16) were observed. (B) Granzyme B plasma levels remained unchanged. Plasma samples were analyzed with a BioPlex 200 reader. Crtl = healthy controls, acute ITP = ITP patients at diagnosis without treatment, acute ITP+IVIg = ITP patients 24-48h after IVIg treatment, and chronic ITP = ITP patients which have a persistent platelet count (<100x 109/L) which lasts longer than one year after initial diagnosis. Statistical analyses were performed using one-way ANOVA followed by multiple comparisons tests to compare the mean ranks between groups. Data are presented as Standard Error of the Mean (SEM). Significance is shown as p < 0.033 (*), p < 0.0021 (**), p < 0.0002 (***), p < 0.001 (****). (TIFF) [file pone.0244848.s002.tiff]

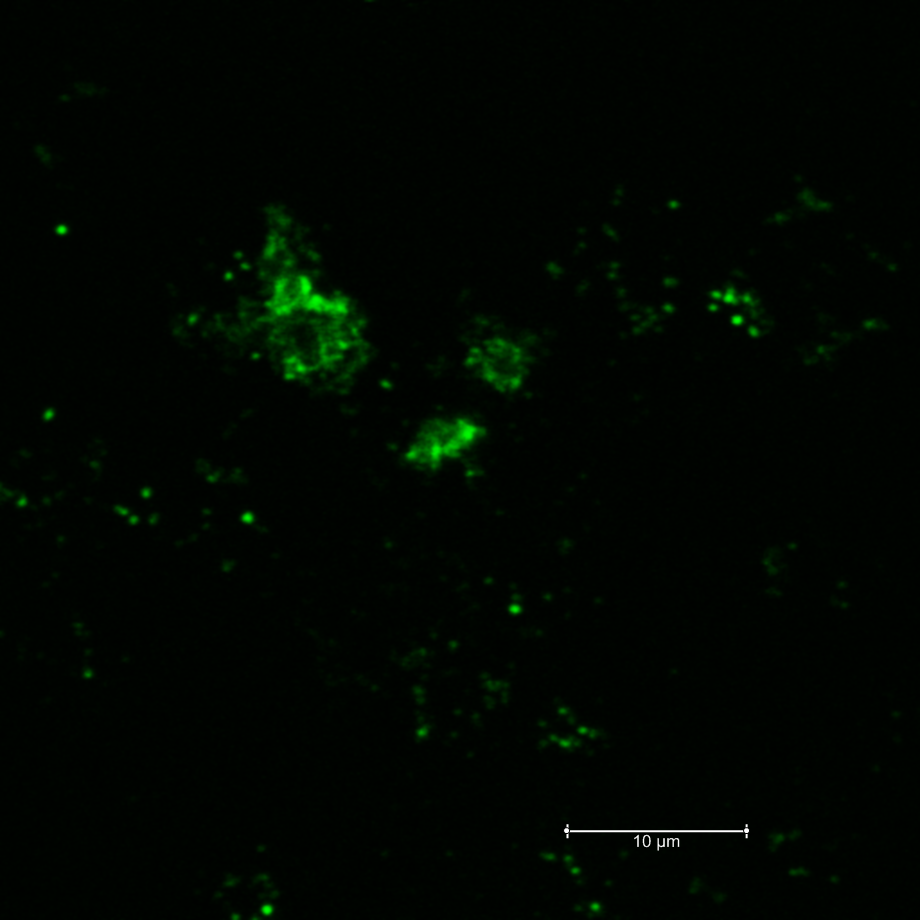

Supplement: S3 Fig — Representative confocal image showing the FADD immunofluorescence staining (green). Shown is one confocal plane, objective 63x glycerol with a numerical aperture of 1.3. Images were processed by using confocal laser scanning microscope SP8 (Leica). Scale bar 10μm. (TIF) [file pone.0244848.s003.tif]

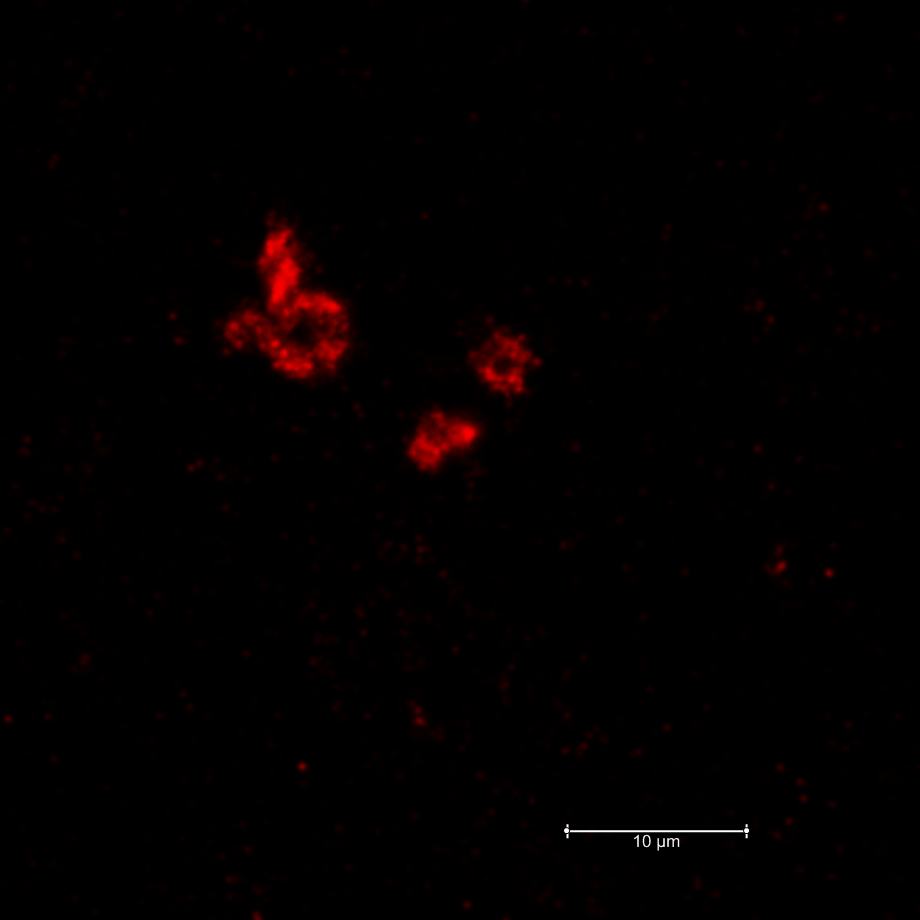

Supplement: S4 Fig — Representative confocal image showing the DJ-1 immunofluorescence staining (red). Shown is one confocal plane, objective 63x glycerol with a numerical aperture of 1.3. Images were processed by using confocal laser scanning microscope SP8 (Leica). Scale bar 10μm. (TIF) [file pone.0244848.s004.tif]

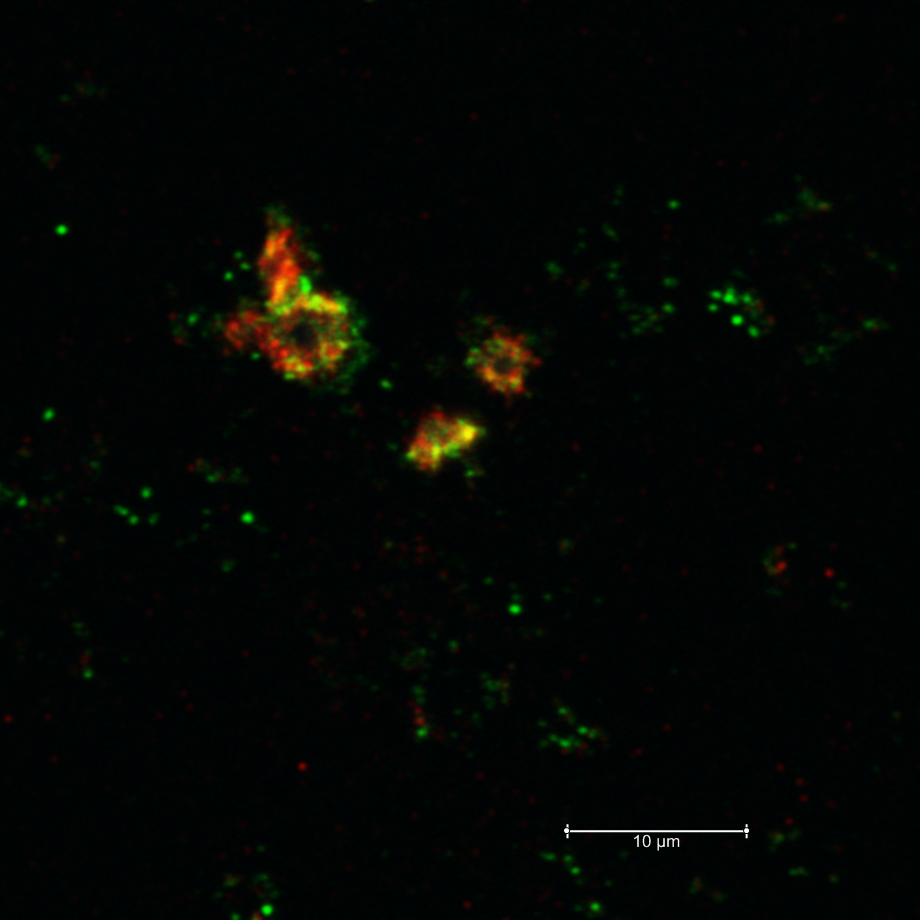

Supplement: S5 Fig — Representative confocal image showing the FADD (green) and DJ-1 immunofluorescence staining (red). Shown is one confocal plane, objective 63x glycerol with a numerical aperture of 1.3. Images were processed by using confocal laser scanning microscope SP8 (Leica). Scale bar 10μm. (TIF) [file pone.0244848.s005.tif]

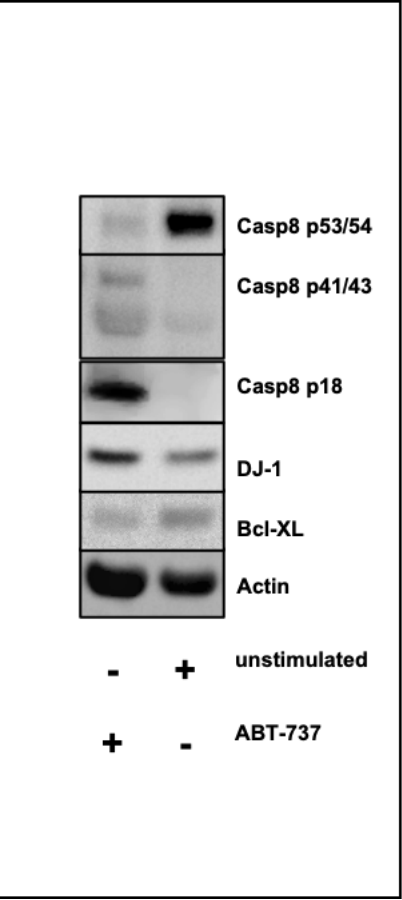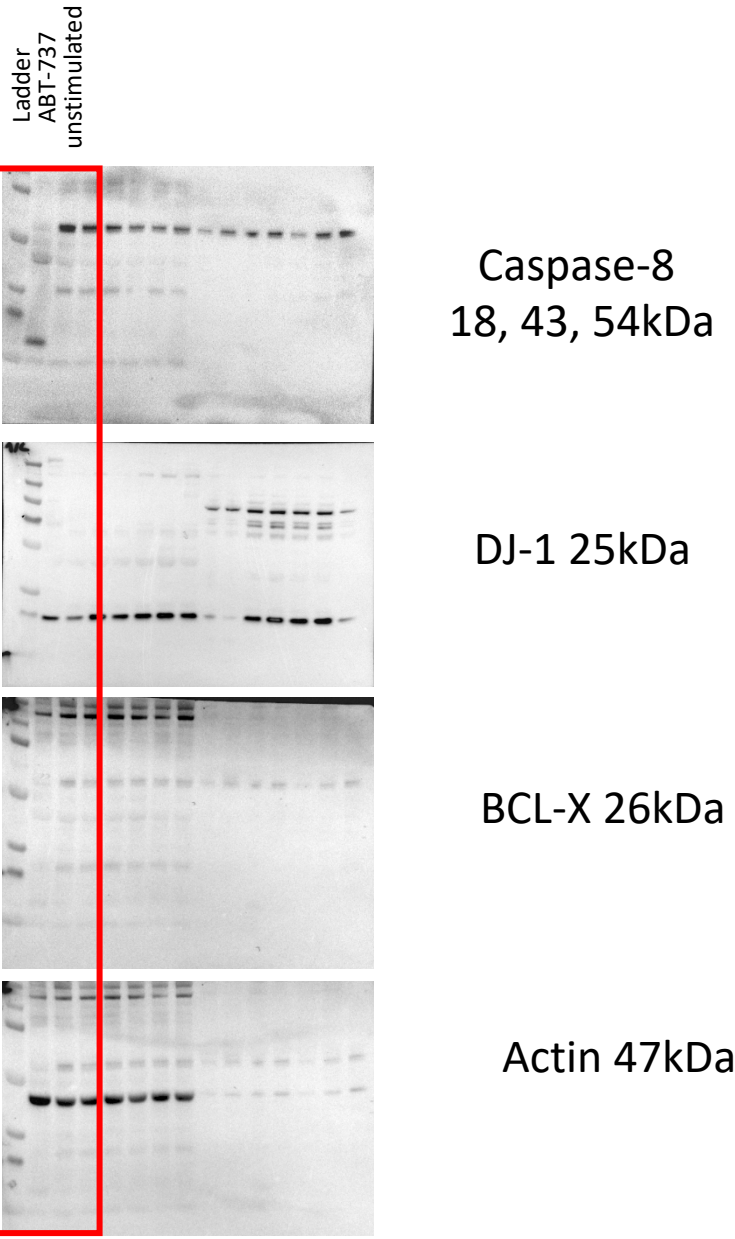

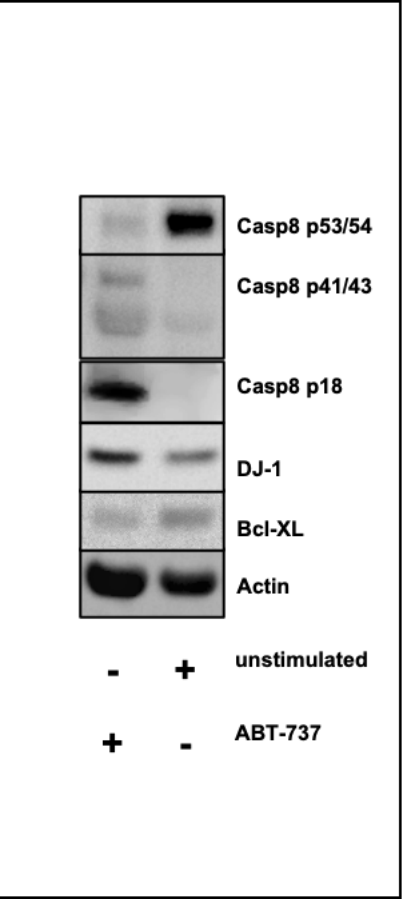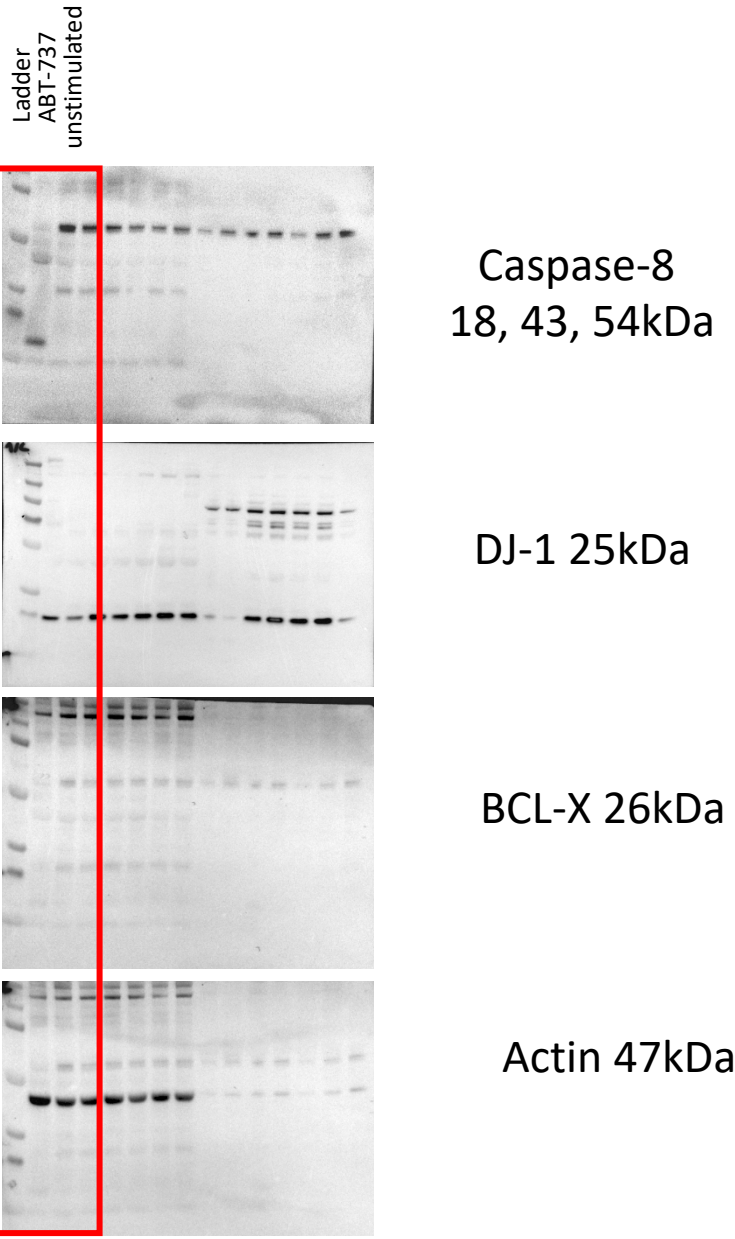

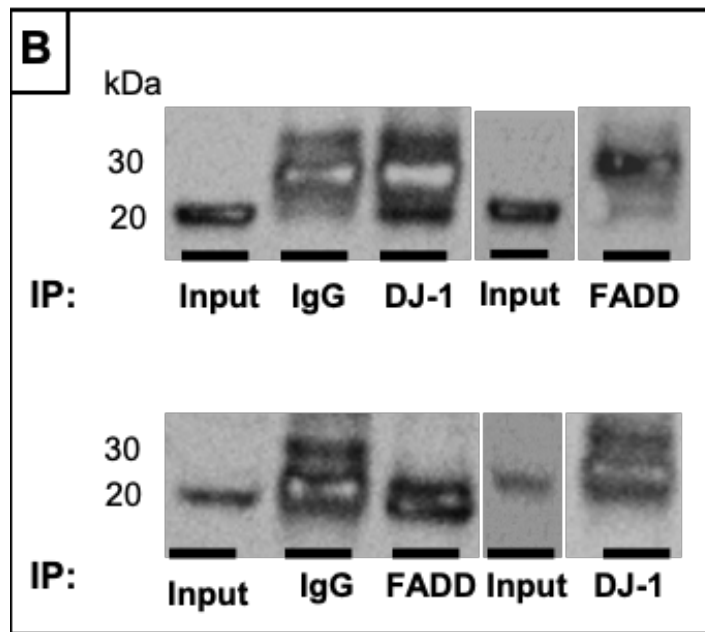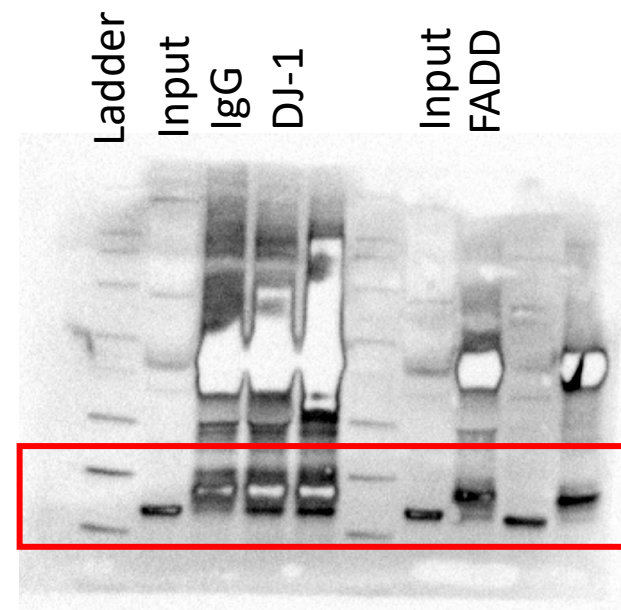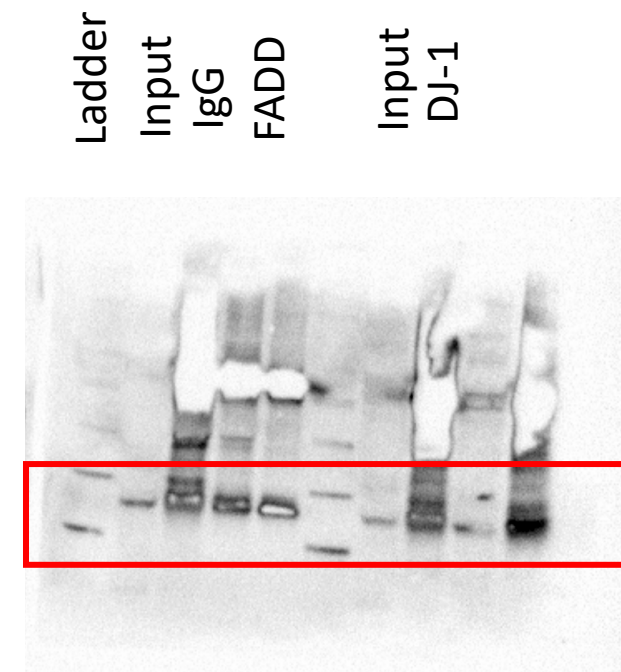

Supplement: S6 Fig — Original blots from Figs 2B and 3C and S2 Fig are shown. (PDF) [file pone.0244848.s006.pdf]
