# Supplementary material for: Platelets express adaptor proteins of the extrinsic apoptosis pathway and can activate caspase-8
Source: PLoS One. 2021 Jan 11;16(1):e0244848. doi: 10.1371/journal.pone.0244848 (PMC7799768; doi:10.1371/journal.pone.0244848)
Supplement: S1 Table — (DOCX) [file pone.0244848.s007.docx]

S1 Table. Antibodies used for flow cytometry, co-Immunoprecipitation, Western blotting and IF staining.

| Name | Fluorochrome | Company | Host | Catalog number |
| --- | --- | --- | --- | --- |
| 1. Antibodies used for flow cytometry analyses and IF staining | | | | |
| CD42a | PERCP | BD Biosciences | mouse (clone: Beb1) | 340537 |
| CD42b | PE | BD Biosciences | mouse (clone: HIP1) | 555473 |
| PARK7/DJ-1* | No conjugate | NovusBio | mouse | NBP1-92715 |
| FADD* | No conjugate | Millipore | rabbit | 341282 |
| TRADD | No conjugate | NovusBio | rabbit | NB100-56169SS |
| c-FLIP | No conjugate | NovusBio | rabbit | NB100-56141 |
| TRAF5 | No conjugate | EnzoLifeSciences | mouse | ALX-804-250-C100 |
| Dedaf | No conjugate | Sigma-Aldrich | rabbit | AB3637 |
| TRAF2 | No conjugate | Gift from Lynn Wong | mouse | - |
| Caspase-8* | No conjugate | Enzo Life Sciences | mouse | ALX-804-242-C100 |
| DAXX* | No conjugate | R&D systems | goat | AF4548 |
| Anti-mouse | Alexa 488 | Abcam | donkey | AB150105 |
| Anti-rabbit | Alexa 488 | Abcam | goat | AB150077 |
| Anti-mouse | Alexa 647 | Abcam | goat | AB150113 |
| 1. Antibodies used for co-Immunoprecipitation and Western Blotting | | | | |
| Bcl-XL | No conjugate | R&D systems | rabbit | AF800 |
| Bid | No conjugate | Cell signaling | rabbit | #2002S |
| Caspase-3 | No conjugate | Cell signaling | mouse | 9668 |
| PARK7/DJ-1 | No conjugate | NovusBio | mouse | NBP1-92715 |
| FADD | No conjugate | BD Pharmigen | mouse | 5037626 |
| Anti-Mouse IgG1 | No conjugate | Thermo Fischer | - | 14-4714-82 |
| Anti-rabbit IgG | No conjugate | Santa Cruz | - | sc-2027 |
| Anti-mouse | HRP | Jackson Laboratories | goat | 115-035-174 |
| Anti-rabbit | HRP | Jackson Laboratories | goat | 111-035-144 |

* Antibodies also used for co-IP or WB.
